# Soil Enzyme Activity in Soils Subjected to Flooding and the Effect on Nitrogen and Phosphorus Uptake by Oilseed Rape

**DOI:** 10.3389/fpls.2019.00368

**Published:** 2019-03-27

**Authors:** Chiming Gu, Shujie Zhang, Peipei Han, Xiaojia Hu, Lihua Xie, Yinshui Li, Margot Brooks, Xing Liao, Lu Qin

**Affiliations:** ^1^Oil Crops Research Institute, Chinese Academy of Agricultural Sciences, Key Laboratory of Biology and Genetic Improvement of Oil Crops, Ministry of Agriculture, Wuhan, China; ^2^Department of Biochemistry and Microbiology, Rhodes University, Grahamstown, South Africa

**Keywords:** waterlogging stress, oilseed rape, soil enzyme activities, available N, available P

## Abstract

Waterlogging presents one of the greatest constraints for agricultural crops. In order to elucidate the influences of waterlogging stress on the growth of oilseed rape, a pot experiment was performed investigating the impact of waterlogging on nitrogen (N) and phosphorus (P) accumulation in oilseed rape, and mineral N and available P profiles and enzyme activities of soils. The experiment included waterlogging treatments lasting 3 (I), 6 (II), and 9 (III) days, and a control treatment without waterlogging (CK). Results showed that waterlogging lasting 3 or more days significantly depressed the growth of oilseed rape, and prolonged the recovery time of plant growth with the period of flooding. Waterlogging notably influenced the N and P concentrations in plant tissues, and also affected mineral N, available P profiles, and activities of enzymes (including urease, phosphatase, invertase, and catalase) in the soils. With the exception of catalase, flooding suppressed the activity of urease, phosphatase, and invertase to varying degrees, and the longer the flooding time, the greater the suppression. The effect of waterlogging on mineral N and P profiles resulted from the altered proportions of NH_4_^+^-N and NO_3_^-^-N, and the decreased available P concentrations in these soils, respectively. The effect on P was more significant than on N in both soil nutrient profile and plant utilization.

## Introduction

Waterlogging is one of the greatest constraints imposed on agricultural crops, affecting both vegetative and reproductive phases of the plant life cycle (Kumutha et al., 2008; Irfan et al., 2010; Shabala, 2011).

Excess water causes a sharp decrease in soil redox potential, resulting in significant changes to the soil elemental profile (Khabaz-Saberi and Rengel, 2010; Shabala, 2011). It also leads to a high partial pressure of CO_2_ in the root zone, resulting in serious consequences for root growth and metabolism (Shabala, 2011). Waterlogging induces oxygen starvation in the soil, arising from an imbalance between the slow diffusion of gases in water compared with air, and the quick rate at which oxygen is consumed by microorganisms and plant roots (Kumutha et al., 2008; Irfan et al., 2010). Since roots and rhizomes are essentially aerobic organs, the cessation of aerobic respiration as a consequence of waterlogging results in a drop in energy level status of root cells, and uptake and transport of ions by the plants decline, which is then fatal.

Soil enzymes are sensitive to the stresses on ecosystems, which in turn affect enzyme activities. They have been suggested as suitable indicators of soil quality because of their intimate relationship with soil biology, the ease with which they can be measured, and their rapid response to change in soil management and environment (García-Ruiz et al., 2009; Burke et al., 2011; Henry, 2012). Soil enzymes can promote the transformation of matter and energy in soil, and the activity of soil enzymes has a close relationship with soil nutrients and their availability. Acid phosphatase, alkaline phosphatase, urease, and arylsulfatase activities have been significantly correlated with redox potential (Eh), and Pulford and Tabatabai (1988) found that waterlogging of soils markedly affected the reaction rates of these enzymes, with possible consequences for nutrient cycling. Waterlogging induces a significant accumulation of organic substances produced as a result of anaerobic metabolism in both plants and rhizosphere microorganisms in soils (Shabala, 2011). These organic substances, such as ethanol, ethylene, acetaldehyde, and various short-chain fatty acids and phenolics, defined as soil phytotoxins, may have significant adverse effects on both plant growth and soil quality (Setter et al., 2009; Shabala, 2011). Therefore, the effects of waterlogging on soil enzyme activity and on soil microbial biomass and activity, in addition to the process of soil redox, result in changes in soil properties that negatively affect crop production as well (Yang et al., 2005; Zhang et al., 2016). Oilseed rape (*Brassica napus* L.) is one of the major crops for the production of oilseed and the second most common edible oil source in the human diet all over the world (FAO, 2016). It is susceptible to the detrimental effects on growth and seed yield caused by waterlogging of the soil (Zhang et al., 2016). In southern China, oilseed rape is planted as a rotation crop after rice has been harvested, which means young seedlings are exposed to the moist paddy soil, and rainfall during the seedling stage leads to temporarily waterlogged soils. This is really problematic for oilseed rape production, due to the sensitivity of oilseed rape to waterlogging stress (Zou et al., 2014). Over recent years, a large amount of information has been accumulated regarding the responses of crop species to waterlogging stress. Many studies report that the growth and yield of crops such as cotton (Bange et al., 2004), wheat (Jiang et al., 2008; Sharma et al., 2010), maize (Souza et al., 2011), and soybean (Rhine et al., 2010) have been significantly depressed by waterlogging stress.

However, there are few reports on the effects on soil enzyme activity and subsequent changes in the soil properties under flooding and the correlation between the flooding and growth of rapeseed seedlings after flooding. Thus, in the present study, we investigated the influences of waterlogging stress on the growth of seedling oilseed rape (*B. napus* L. cv. Zhongshuang No. 11), as well as the effect of waterlogging on soil enzyme activity, nitrogen (N) and phosphorus (P) accumulation, and soil profiles, with the aim of elucidating the relationships between them, through a pot experiment.

## Materials and Methods

### Soil Collection and Preparation

The experiment was conducted from October to December 2011 at Oil Crops Research Institute of the Chinese Academy of Agricultural Sciences, Wuhan, China (114°20′ E, 30°37′ N). The monthly average air temperature for October, November, and December in 2011 was 16.0, 9.7, and 4.2°C, respectively. Yellow-brown soil (30.6% clay, 35.3% silt, 34.1% sand) used in the present study was taken from the cultivated layer (0–20 cm) of farmland in Yangluo town, Wuhan city, Hubei province, China (114°54′ E, 30°37′ N), and had the following chemical characteristics: pH (H_2_O: soil = 5:1) was 6.71, dissolved organic carbon (DOC) was 104.1 mg kg^-1^, total nitrogen was 1.28 g kg^-1^, NO_3_^-^-N was 5.4 mg kg^-1^, NH_4_^+^-N was 4.4 mg kg^-1^, available phosphorus was 36.2 mg kg^-1^, and available potassium was 63.9 mg kg^-1^. The soil was air-dried, passed through a 2 mm mesh sieve, and placed in pots (5.0 kg pot^-1^). All pots were arranged under a rain shelter with natural sunshine (12 h daily photoperiod) and ambient temperature, and the moisture content of the soil was adjusted to approximately 70% water holding capacity with deionized water.

### Plant Culture

Seeds of winter oilseed rape (*B. napus* L. cv. Zhongshuang No. 11), a double-low rapeseed variety widely cultivated in Yangtze River Basin, China, were rinsed several times with deionized water after being disinfected in 1% sodium hypochlorite solution for 10 min to eliminate possible seed-borne microorganisms, and dried at room temperature (20–22°C) before being sown. Fertilizer was applied to each pot at the beginning of the experiment to ensure uniform nutrient availability, with urea (0.75 g N), superphosphate (0.5 g P_2_O_5_), potassium chloride (0.75 g K_2_O), and borax (0.15 g B). Each pot was watered daily with deionized water to complement loss by evaporation, which was measured by weighing. The seeds were sown on 1 October 2011, and thinned to one plant per pot by hand when the seedlings had fully developed four true leaves. Plant protection was applied as needed.

### Experimental Design

Waterlogging treatments were carried out at the five true leaves stage. Pots comprising the present study, including waterlogging treatments lasting 3 days (treatment I), 6 days (treatment II), 9 days (treatment III), and a no waterlogging control (approximately 70% water holding capacity, CK), were completely randomly arranged under the same rain shelter as described above. For the waterlogging treatments, pots were flooded with a 1 cm water layer over the soil surface, and allowed to drain and return to the previous water regime after the period of flooding.

### Sampling and Analysis

At 0, 3, 6, 9, 12, 15, and 18 days from the beginning of the waterlogging treatments, plants were destructively sampled in five replicates from each of the treatments. Soil samples were sampled from the surface layer (0–5 cm) of each pot, kept at 4°C and assayed as soon as possible for mineral nutrient concentrations and soil enzyme activity. Each plant was separated carefully into roots and shoots and oven dried at 70°C for 48 h to constant weight for biomass determination. For further analysis, samples were milled to fine powder. Total N and P concentration of each fraction was determined by using a Kjeldahl nitrogen analyzer and Mo-Sb colorimetry methods, respectively. Soil samples were taken from each pot and mineral N (NO_3_^-^-N and NH_4_^+^-N) concentrations were determined by extracting soil samples with 2 M KCl (1:10 soil:extractant) (Bao, 2000) and the extracts analyzed on a flow injection analyzer (AA3-A001-02E, Bran Luebbe, Inc., United States). Soil available P was extracted from 5 g soil samples with 100 ml 0.5 M sodium bicarbonate at pH 8.5 and the mixtures were shaken for 30 min and filtered through pore size 2.5 μm. Colorimetric determination (absorbance at wavelength 700 nm) of phosphate in solution was based on the molybdate-ascorbic acid method (Murphy and Riley, 1962). Total N concentrations in plant tissues were determined by micro-Kjeldahl after being digested in concentrated H_2_SO_4_ with H_2_O_2_ (Zhang et al., 2016). Total phosphorus concentrations in plant tissues were determined by the following method: the samples were digested by adding the concentrated sulfuric acid and hydrogen peroxide, then the digestion solution reacted with ammonium molybdate-antimony potassium tartrate solution to form a blue complex, and total phosphorus was determined by spectrophotometry at wavelength of 700 nm (Liu et al., 2011).

### Soil Enzyme Activity Analysis

Soil enzyme activities were analyzed in the fresh soil samples and were assayed as described by Tabatabai (1994). Soil phosphatase activity (mg Phenol kg^-1^ h^-1^) was estimated by determination of the phenol release after incubation of samples with phenyl disodium phosphate (0.5%) for 2 h at 37°C. Urease activity (mg NH_4_^+^ kg^-1^ h^-1^) was measured by determination of the NH_4_^+^ released in the hydrolysis reaction after incubation of samples with urea (1%) for 3 h at 38°C. Invertase activity (mg glucose kg^-1^ h^-1^) was measured by determination of the glucose released in the hydrolysis reaction after incubation of samples with sucrose (8%) for 3 h at 37°C. Soil catalase activity (mg KMnO_4_ kg^-1^) was determined by measuring the reduction in H_2_O_2_ by titration with 0.1 M KMnO_4_ after having shaken a 5 g soil sample in 100 ml distilled water for 30 min.

### Statistical Analysis

All data are presented as the mean value of five replicates. Analyses were performed using a SPSS statistical software package (Version 16.0) and the variance (*P* < 0.05) of the data was analyzed by one-way ANOVAs test (Duncan’s test).

## Results

### Plant Dry Weight

**FIGURE 1:**
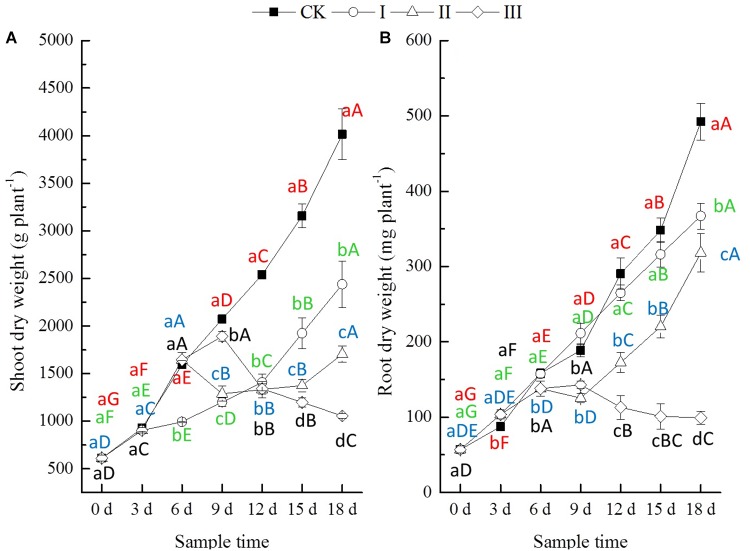
Dry weight (DW, mg of plant) shoot **(A)** and root **(B)**. Error bars represent standard error (*n* = 5). CK, I, II, and III denote control with no waterlogging, waterlogging lasting 3, 6, and 9 days, respectively. Different lowercase letters represent significant differences in values among treatments at the same sample time. Different capital letters represent significant differences in values in specific treatments over the entire experimental period. Red letters represent CK, while green, blue, and black letters represent treatments I, II, and III, respectively (hereinafter).

During the period of flooding, the differences in shoot dry weights of winter oilseed rape seedlings among treatments were not significant until the period of flooding lasted for 6 days. The root dry weight was significantly decreased at the period of flooding lasting 9 days (treatment III), while the shoot dry weight decreased significantly after a significant increase (Figure 1). At the end of flooding, compared with CK, the shoot dry weight of plants from treatment I decreased slightly, by 2.2% (at the third day), that from treatment II increased slightly, by 3.2% (at the sixth day), and that of treatment III decreased significantly by 8.8% (at the ninth day). However, the root dry weights of plants from treatment I increased significantly by 18.2% (at the third day), and those of treatment II (at the sixth day) and III (at the ninth day) decreased significantly by 12.8 and 24.2%, respectively (Figure 1). After the period of flooding, both the shoot dry weight and the root dry weights of treatment I increased significantly with time. The shoot and root dry weights of treatment II decreased continuously for 3 days followed by a continuous increase. The shoot dry weight of treatment III decreased significantly with time, and the root dry weight decreased continuously for 3 days, followed by a stable trend (Figure 1). At the end of this experiment, compared with CK, the dry weights of shoots from treatment I, II, and III showed a significant decrease of 39.3, 57.5, and 73.7%, respectively, and the dry weights of roots from treatment I, II, and III showed a significant decrease of 25.5, 35.3, and 79.8%, respectively (Figure 1).

### Nitrogen and Phosphorus Concentrations in Seedling Winter Oilseed Rape

The effect of waterlogging on the nitrogen (N) and phosphorus (P) concentration of seedling winter oilseed rape among different treatments became more and more significant with time, while both the N and P concentrations of CK showed no significant changes throughout the experiment (Table 1).

**Table 1:** Nitrogen and phosphorus concentration (mg g^-1^ DW) in juvenile winter oilseed rape.

Element	Sample time	Leaf				Root			
		CK	I	II	III	CK	I	II	III
N (mg g^-1^ DW)	0 day	44.3 ± 0.4a	44.3 ± 0.4a	44.3 ± 0.4a	44.3 ± 0.4a	37.1 ± 1.1a	37.1 ± 1.1a	37.1 ± 1.1a	37.1 ± 1.1a
	3 days	43.5 ± 1.7a	45.7 ± 1.2a	45.7 ± 1.2a	45.7 ± 1.2a	38.5 ± 0.8a	40.1 ± 1.2a	40.1 ± 1.2a	40.1 ± 1.2a
	6 days	43.8 ± 0.7b	46.1 ± 1.1a	40.6 ± 1.5b	40.6 ± 1.5b	37.8 ± 0.6b	41.6 ± 0.7a	42.4 ± 1.5a	42.4 ± 1.5a
	9 days	43.6 ± 0.9a	44.1 ± 0.8a	36.2 ± 1.1b	34.3 ± 0.5c	37.4 ± 1.2c	40.1 ± 1.3b	43.2 ± 1.4a	44.8 ± 1.6a
	12 days	44.0 ± 1.3a	44.2 ± 2.1a	37.1 ± 0.8b	34.3 ± 1.1c	38.1 ± 0.5c	39.5 ± 0.8c	43.7 ± 0.6b	46.3 ± 0.7a
	15 days	43.6 ± 2.1a	43.5 ± 1.7a	39.2 ± 0.9b	35.5 ± 1.7c	37.5 ± 0.6c	39.3 ± 0.3c	42.5 ± 0.9b	45.5 ± 1.5a
	18 days	43.9 ± 1.1a	43.7 ± 0.5a	41.6 ± 1.4a	37.1 ± 1.5b	37.9 ± 0.9c	38.7 ± 0.7c	41.8 ± 0.9b	44.6 ± 1.3a
P (mg g^-1^ DW)	0 day	13.7 ± 0.3a	13.7 ± 0.3a	13.7 ± 0.3a	13.7 ± 0.3a	13.8 ± 0.4a	13.8 ± 0.4a	13.8 ± 0.4a	13.8 ± 0.4a
	3 days	13.7 ± 0.2a	13.1 ± 0.1b	13.1 ± 0.1b	13.1 ± 0.1b	13.5 ± 0.7a	11.7 ± 0.5b	11.7 ± 0.5b	11.7 ± 0.5b
	6 days	13.7 ± 0.3a	11.3 ± 0.6b	9.3 ± 0.8c	9.3 ± 0.8c	13.7 ± 0.6a	11.9 ± 0.5b	11.7 ± 0.6b	11.7 ± 0.6b
	9 days	13.5 ± 0.4a	11.8 ± 0.4b	10.0 ± 0.1c	7.0 ± 0.9d	14.0 ± 0.1a	9.5 ± 0.2b	9.3 ± 0.2b	10.1 ± 0.4b
	12 days	13.6 ± 0.4a	12.0 ± 0.6b	9.9 ± 0.2c	7.2 ± 0.5d	14.1 ± 0.2a	8.1 ± 0.5b	8.3 ± 0.3b	8.3 ± 0.3b
	15 days	13.9 ± 0.7a	12.1 ± 0.3b	9.8 ± 0.3c	8.0 ± 0.3d	14.0 ± 0.6a	8.7 ± 0.2b	7.9 ± 0.1c	7.9 ± 0.4c
	18 days	13.6 ± 0.4a	12.2 ± 0.7b	9.6 ± 0.1c	8.3 ± 0.3d	14.5 ± 0.3a	9.1 ± 0.3b	7.0 ± 0.3c	7.1 ± 0.4c


*Means with different letters in a line denote a significant difference between treatment groups according to Duncan’s test (*P* < 0.05). DW denotes plant dry weight (hereinafter).*

During the period of flooding, the concentration of N in the leaves of plants from waterlogging treatments increased slightly in the first 3 days and then decreased significantly. The roots exhibited a significant increase in the concentration of N and then a period of decrease in each of the treatments during the experiment period. Both the leaf and root P concentration of plants from waterlogging treatments decreased significantly with time (Table 1).

At the end of flooding, compared with CK, the leaf N concentration of plants from treatment I increased by 5.1% but those from treatment II and III decreased by 7.4 and 21.3%, respectively, and the root N concentration of treatment I, II, and III increased by 4.2, 12.2, and 19.8%, respectively (Table 1). The leaf P concentration of plants from treatment I, II, and III decreased by 4.4, 32.1, and 48.1%, the root P concentration of plants from treatment I, II, and III decreased by 13.3, 14.6, and 27.9% at the end of flooding, respectively (Table 1). After the flooding period, the leaf N concentration of plants from treatment I decreased, approaching close to the level of those from CK, while the equivalent of treatment II and III increased with time but were still lower than CK. In this period, the root N concentration of plants from treatment I decreased to the CK levels, while the equivalent of treatment II and III increased continuously for 6 days followed by a 6-day decrease. The root N concentration of plants from treatment II and III was significantly higher than that of plants from CK at the end of the experiment (Table 1).

The leaf P concentration of plants from treatment I and II increased, but that of treatment III decreased with time and was significantly lower than that of CK; the root P concentration of plants from the waterlogging treatments decreased significantly with time (Table 1).

At the end of the experiment, compared with CK, the leaf N concentration of plants from treatment I, II, and III decreased by 0.5, 5.2, and 15.5%, the root N concentration of plants from treatment I, II, and III increased by 4.7, 10.3, and 17.7%; the leaf P concentration of plants from treatment I, II, and III decreased by 2.1, 29.4, and 39.0%; while the root P concentration of plants from treatment I, II, and III decreased by 37.2, 51.7, and 51.0%, respectively (Table 1).

### Soil Mineral Nitrogen and Available Phosphorus Concentrations

Soil mineral N (both NO_3_^-^-N and NH_4_^+^-N) and available P concentration of each treatment is shown in Table 2. Soil nitrogen, mainly in the form of NO_3_^-^-N, exists in the soil throughout the experiment. In the course of the experiment, the differences in both mineral N and available P content of the soil in CK were not significant (Table 2). In the waterlogging treatments, soil NH_4_^+^-N concentrations increased significantly in the flooding period and then significantly decreased, while the NO_3_^-^-N and available P content of the soil decreased significantly in the flooding period (Table 2). At the end of the flooding, compared with CK, the NH_4_^+^-N concentration of soil from treatment I, II, and III increased by 66.3, 63.7, and 36.4%, and the NO_3_^-^-N concentration of soil from treatment I, II, and III decreased by 26.1, 38.8, and 59.7%, respectively. The available P concentrations of soil from treatment I, II, and III decreased by 21.2, 36.3, and 35.2%, respectively (Table 2).

**Table 2:** Soil mineral nitrogen and available phosphorus concentrations (mg g^-1^ DW).

Soil nutrient	Sample time	CK	I	II	III
NH_4_-N (mg g^-1^ DW)	0 day	3.55 ± 0.11a	3.55 ± 0.11a	3.55 ± 0.11a	3.55 ± 0.11a
	3 days	3.35 ± 0.10b	5.57 ± 0.22a	5.57 ± 0.22a	5.57 ± 0.22a
	6 days	3.58 ± 0.16c	6.47 ± 0.19a	5.86 ± 0.20b	5.86 ± 0.20b
	9 days	3.76 ± 0.21c	4.91 ± 0.13b	5.62 ± 0.18a	5.13 ± 0.20b
	12 days	3.80 ± 0.09d	4.52 ± 0.17c	5.32 ± 0.14b	6.23 ± 0.07a
	15 days	3.53 ± 0.05d	3.94 ± 0.08c	5.12 ± 0.33a	5.71 ± 0.16b
	18 days	3.48 ± 0.05c	3.49 ± 0.15c	3.94 ± 0.05b	5.18 ± 0.14a
NO_3_-N (mg g^-1^ DW)	0 day	4.91 ± 0.09a	4.91 ± 0.09a	4.91 ± 0.09a	4.91 ± 0.09a
	3 days	4.83 ± 0.18a	3.57 ± 0.09b	3.57 ± 0.09b	3.57 ± 0.09b
	6 days	4.87 ± 0.06a	4.99 ± 0.08a	2.98 ± 0.15b	2.98 ± 0.15b
	9 days	4.72 ± 0.08a	4.97 ± 0.10a	3.14 ± 0.12b	1.90 ± 0.05c
	12 days	4.80 ± 0.12b	5.15 ± 0.03a	3.62 ± 0.05c	2.28 ± 0.13d
	15 days	4.75 ± 0.06a	4.83 ± 0.07a	3.79 ± 0.09b	2.54 ± 0.08c
	18 days	4.79 ± 0.05a	4.72 ± 0.18a	4.13 ± 0.16b	3.10 ± 0.15c
Available P (mg g^-1^ DW)	0 days	38.2 ± 1.5a	38.2 ± 1.5a	38.2 ± 1.5a	38.2 ± 1.5a
	3 days	37.3 ± 0.5a	29.4 ± 0.8b	29.4 ± 0.8b	29.4 ± 0.8b
	6 days	37.5 ± 0.4a	30.3 ± 1.6b	23.9 ± 0.4c	23.9 ± 0.4c
	9 days	36.9 ± 1.4a	31.5 ± 0.9b	28.0 ± 0.3c	23.9 ± 0.6d
	12 days	38.1 ± 1.6a	33.2 ± 0.6b	28.7 ± 1.3c	25.4 ± 1.7c
	15 days	36.6 ± 1.0a	35.8 ± 0.7a	32.5 ± 0.8b	25.8 ± 1.1c
	18 days	36.8 ± 0.6a	36.1 ± 1.0a	34.8 ± 0.7b	28.3 ± 0.7c


*Means with different letters in a line denote a significant difference between treatment groups according to Duncan’s test (*P* < 0.05). DW denotes plant dry weight.*

After the period of flooding, the soil from waterlogging treatments showed a trend of decreasing NH_4_^+^-N concentrations, but the NO_3_^-^-N and available P concentrations increased significantly. At the end of the experiment, compared with CK, the NH_4_^+^-N concentrations of soils from treatment I, II, and III increased by 0.3, 13.2, and 48.9%, and the NO_3_^-^-N concentrations decreased by 1.5, 13.8, and 35.3%, respectively, while the available P concentrations of soil from treatment I, II, and III were decreased by 1.9, 5.4, and 23.1% (Table 2).

### Soil Enzyme Activities

**FIGURE 2:**
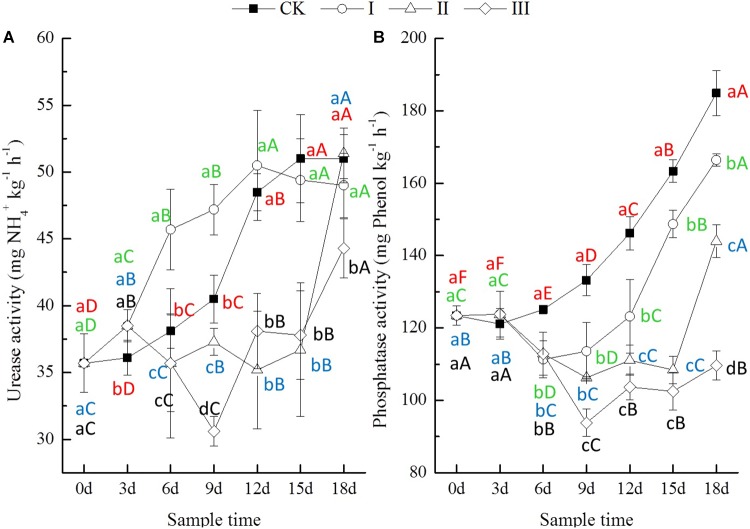
Urease (**A**, mg NH_4_^+^ kg^-1^ h^-1^) and phosphatase (**B**, mg phenol kg^-1^ h^-1^) activity of soils.

**FIGURE 3:**
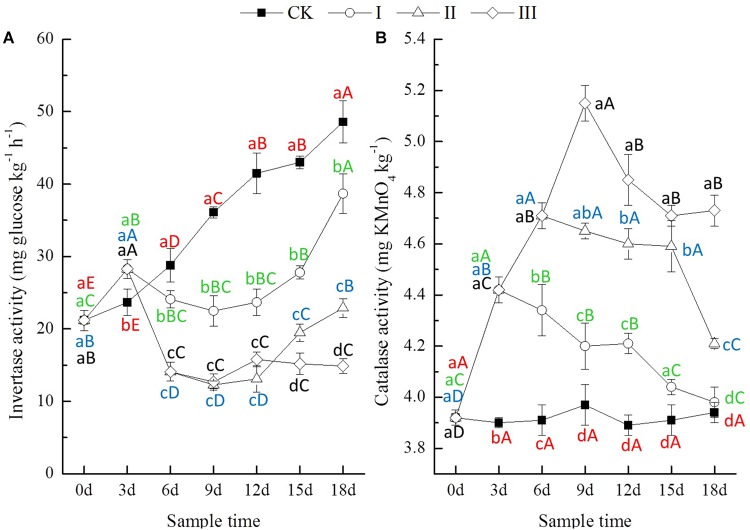
Invertase (**A**, mg glucose kg^-1^ h^-1^) and catalase (**B**, mg KMnO_4_ kg^-1^) activity of soils.

The activities of soil enzymes, including urease, phosphatase, invertase, and catalase, were significantly influenced by waterlogging, and the influences varied depending on how long the period of flooding lasted (Figure 2, 3). During the experimental period, the urease activity in treatment I increased significantly, while in treatment II and III, it showed a significant decrease during flooding until the 12th day (Figure 2A). The phosphatase activity of soil from the flooding treatments was stable for the first 3 days and then decreased significantly until the 9th day (Figure 2B), while both the urease and phosphatase activities of soil from CK increased significantly throughout the whole experiment period (Figure 2). At the end of flooding, the urease activity of soil from treatment I increased by 6.6%, while that of treatment II and III decreased by 6.3 and 24.4%; the phosphatase activity of soil from treatment I increased by 2.2%, and that of soil from treatment II and III decreased by 9.8 and 29.6%, respectively (Figure 2).

After the period of flooding, the urease activity of soil from the waterlogging treatments increased significantly with time. However, the phosphatase activity of soil from treatment I and II decreased continuously until 3 days later, but that from treatment III increased after the flooding period (Figure 2). At the end of the experiment, compared with CK, the urease activity of soil from treatment I and III decreased by 3.9 and 13.1%, respectively, while that from treatment II increased by 0.8%. The phosphatase activity of soil from treatment I, II, and III decreased significantly by 10.0, 22.1, and 40.7%, respectively (Figure 2).

The soil invertase activity of CK increased significantly with time, but there were no significant differences in soil catalase activity throughout the experiment (Figure 3). During the period of flooding, the invertase activity of soil from waterlogging treatments showed a significant decrease after increasing significantly at the flooding lasting 3 days. The catalase activity of soil from waterlogging treatments increased significantly with time. At the end of flooding, compared with CK, the invertase activity of soil from treatment I increased by 19.4% but that of treatment II and III decreased by 51.0 and 64.8%; the catalase activity of soil from treatment I, II, and III increased by 13.3, 20.5, and 29.7%, respectively (Figure 3). After the period of flooding, the invertase activity of soil from treatment I and II increased significantly with time after decreasing continuously for 3 days, while that of treatment III increased slightly. The catalase activity of soil from waterlogging treatments decreased significantly with time, but only that of treatment I was close to the activity levels in soil from CK at the end of the experiment (Figure 3). At the end of the experiment, compared with CK, the invertase activity of soil from treatment I, II, and III decreased by 20.3, 52.9, and 69.3%, while the catalase activity of soil from treatment I, II, and III increased by 1.0, 6.8, and 20.1%, respectively (Figure 3).

## Discussion

Many studies have found that oilseed rape appears not to be able to overcome the detrimental effect of waterlogging on its growth and seed yield. For example, Boem et al. (1996) reported that the seed yield of hybrid winter oilseed rape (cv. Iciola 41) was significantly decreased due to waterlogging of 3 or more days, and the yield declined more in winter flooding than in spring. Wollmer et al. (2017) also found that exposures of oilseed rape (*B. napus* L.) to waterlogging at seedling and floral bud stage significantly decreased yield by 21 and 13%, respectively. The present study produced the same results, showing that waterlogging lasting for 3 or more days significantly depressed the growth of seedling winter oilseed rape (*B. napus* L. cv. Zhongshuang No. 11) (Figure 1). The results indicated that the effects of waterlogging stress on seedling winter oilseed rape growth could be attributed mainly to its influence on the process of growth recovery after flooding, which recovery was delayed for the period that waterlogging lasted.

During the period of flooding, the leaf nitrogen (N) concentrations were significantly decreased, but the root N concentrations increased significantly with time, while both leaf and root phosphorus (P) concentrations decreased significantly with time in the present study (Table 1). Zhou et al. (1997) found that, after subjecting winter oilseed rape (*B. napus* L. cv. 601) seedlings to waterlogging for 30 days, the leaf N concentrations decreased by 7.73%, but the root N concentrations increased by 3.40%, and our research results were consistent with this study. Milroy et al. (2009) found through a field experiment that the N and P concentrations of cotton leaves (*Gossypium hirsutum* L.) were significantly decreased by waterlogging. Waterlogging blocks the oxygen supply to the roots, inhibiting root respiration and resulting in a severe decline in energy status of root cells, causing a dramatic decline in uptake and transport of ion in plants (Visser et al., 2003; Kumutha et al., 2008; Irfan et al., 2010). Those studies indicated that waterlogging stress significantly influenced the assimilation of mineral elements in seedling winter oilseed rape.

Waterlogging causes a sharp decrease in soil redox potential and leads to high partial pressure of CO_2_ in the root zone (Greenway et al., 2006; Khabaz-Saberi and Rengel, 2010; Shabala, 2011), which results in substantial changes to the soil elemental profile and the plant elemental assimilation. In the present study, waterlogging significantly increased the NH_4_^+^-N concentration and significantly decreased the NO_3_^-^-N concentration of soil during the period of waterlogging (Table 2). These results indicate that the predominant effect of waterlogging on the soil mineral N profile is the alteration of the proportion of NH_4_^+^-N and NO_3_^-^-N (Table 2). Because NH_4_^+^-N is dominant in waterlogged soil, and the assimilation and accumulation of NH_4_^+^-N is mainly at plant roots (Cao et al., 2008), this leads to the absorption and assimilation of NO_3_^-^-N being repressed in waterlogged soils (Dan and Brix, 2009; Jampeetong and Brix, 2009; Shabala, 2011). This is the reason why the concentration of leaf N decreased, while the root N concentration increased under waterlogging stress in the present study (Table 1).

Plant uptake, redox conditions, and phosphatase can affect the phosphorus adsorption and desorption characteristics of soil. Zhang et al. (2016) found that cultivation with waterlogging could reduce greenhouse soil available P content, and they suggested that this might be caused by plant uptake on soil available P. In contrast to our research, (Liang, 1996) found that under flooding conditions, Eh gradually decreased, and with prolonged flooding time, the content of available P in soil increased markedly. After flooding, Eh rose slightly and the soil available P decreased by about 2.8 times (Liang, 1996). Phosphatase plays a big role in the process of alteration of P components; it catalyzes the hydrolysis of phosphate or phosphoric anhydride, and its activity directly affects the decomposition and transformation of soil organic P and its bioavailability (Allison et al., 2007). In our study, there was a continuous decrease in P during the flooding period, and this novel result might be due both to uptake of P by oilseed rape, and to lower phosphatase activity. Lower phosphatase activity would decrease the available P concentrations of soils (Table 2) and therefore lower P content in seedling winter oilseed rape tissues (Table 1).

The results in this study indicated that, evidenced by the soil mineral P profile, the main effect of waterlogging on P was to decrease its availability. After the period of flooding was terminated, both N and P concentrations of plant tissues recovered to the CK levels, but their recovery times were prolonged with the length of the period of waterlogging. In addition, the recovery times of N were shorter than those of P (Table 1), because the recovery rates of available P were lower than those of mineral N (NO_3_^-^-N and NH_4_^+^-N) in soils (Table 2), and the assimilation pathways of N and P differ in seedling winter oilseed rape (Li et al., 2015).

Soil enzyme activities are indicators of soil microbial status and soil physico-chemical conditions (Sardans et al., 2008; García-Ruiz et al., 2009; Burke et al., 2011; Henry, 2012). Soil enzyme activities are mainly limited by edaphic factors such as soil pH, availability of C, nutrients, and the water profile, as well as the interactions between them (Wang et al., 2009; Fernández-Calviño et al., 2010), and they are important in catalyzing several reactions necessary for nutrient cycling in soils. As the first phase of N mineralization and an indispensable step for N uptake by plants, the activity of urease releases NH_4_^+^-N through urea hydrolysis and is essential in the chain of hydrolysis of amino compounds (Sardans et al., 2008; Tao et al., 2009). Phosphatase activity is linked to the potential to mineralize P in soils and plays a critical role in soil P cycles, which is correlated with P stress and plant growth (Tao et al., 2009). In the present study, the urease and phosphatase activities were increased at 3 days and then decreased significantly with time during the period of flooding. After the period of flooding, the activities of urease and phosphatase increased significantly with time (Figure 2). These results might explain the seedling winter oilseed rape growth (Figure 1) and the nutrients (N and P) availability of soils (Table 2).

Invertase catalyzes the hydrolysis of sucrose into glucose and fructose, and its activity is linked to the soil microbial biomass (Tao et al., 2009). In the present study, the invertase activity increased significantly at 3 days and then decreased significantly for 3 days during the period of flooding, followed by an increase with time after flooding (Figure 3A). This result might indicate that waterlogging influenced the communications and activities of soil microorganisms. It is well known that the products of oxidation–reduction reactions, such as hydrogen peroxide, superoxide radicals, and hydroxyl radicals, are highly toxic for cells and might damage cellular macromolecules. Catalase can split hydrogen peroxide into molecular oxygen and water and thus prevent cell damage by reactive oxygen species (Stȩpniewska et al., 2009). The catalase activity increased significantly with the period of flooding and decreased significantly with time after flooding in the present study (Figure 3B). This result suggested that waterlogging induced oxidation of the rhizosphere of the seedling winter oilseed rape, and the extent of oxidative stress increased with the length of the period of flooding, and none of the soil enzymes returned to their former activity levels, even after 18 days, after being waterlogged for 9 days (Figure 3).

## Conclusion

The present study showed that waterlogging lasting 3 or more days significantly depressed the growth of seedling winter oilseed rape (*B. napus* L. cv. Zhongshuang No. 11). The effects of waterlogging stress were mainly attributed to the influence of waterlogging on the growth recovery process after flooding, and the growth recovery process was prolonged with the length of the period of waterlogging. Waterlogging significantly changed the proportion of NH_4_^+^-N and NO_3_^-^-N of soil, and thus influenced the accumulations of N in plant tissues. Soil phosphatase activity was significantly decreased with waterlogging, resulting in decreased soil available P content and thus the amount of P accumulation in the plants. Waterlogging can influence the communications and activities of soil microorganisms, evidenced in this study by change in invertase activity, and particularly by increased catalase activity in response to the oxidation of the rhizosphere induced by waterlogging.

## Author Contributions

CG, LX, and LQ contributed to conceptualization. SZ, CG, and LQ contributed to data curation. LX and XH contributed to formal analysis and project administration. LQ and XL contributed to funding acquisition and resources. PH, SZ, and YL contributed to investigation. SZ and XL contributed to methodology. YL, PH, and MB were responsible for software. CG, XL, LQ, and MB contributed to supervision. CG, YL, and XL contributed to validation. CG, XL, and LQ contributed to visualization. CG and SZ contributed to writing the original draft. CG, LQ, and MB contributed to writing, review, and editing.

## Conflict of Interest Statement

The authors declare that the research was conducted in the absence of any commercial or financial relationships that could be construed as a potential conflict of interest.
